# Let’s Google It: Assessing the Readability of ADHD-Related Terms in Google Searches

**DOI:** 10.1192/j.eurpsy.2026.11343

**Published:** 2026-07-31

**Authors:** K. Petersen, C. Noureddine, C. Wang, A. Sterchele, S. Vendidandi, A. Guo

**Affiliations:** 1 Psychiatry, Icahn School of Medicine at Mount Sinai, New York; 2 Rutgers University, New Brunswick, United States

## Abstract

**Introduction:**

Health literacy is a critical determinant of healthcare access and outcomes, as limited health literacy can pose a significant barrier for individuals seeking to understand conditions such as attention-deficit hyperactivity disorder (ADHD).

**Objectives:**

This study aims to compare the readability levels of online information related to ADHD, ADD, and ADHD medications, and to identify opportunities to improve the accessibility of ADHD-related content to empower patients to make informed decisions.

**Methods:**

The first 20 Google searches were analyzed for each: “ADHD,” “ADD”, “attention-deficit/hyperactivity disorder,” “attention deficit disorder,” “Ritalin,” “Concerta,” and “Adderall.” Texts were evaluated for word count, complex words, syllables per word, and letters per word using the Good Calculators Flesch Kincaid Calculator. Readability was measured using Flesch-Kincaid Reading Ease (FKRE) and Flesch-Kincaid Grade Level (FKGL) scores.

**Results:**

A one-way ANOVA revealed significant differences in FKGL (*F* = 5.62, *p* < .001). The effect size was large (Cohen’s *f* = 0.5; η^2^ = 0.20), with diagnosis/treatment type accounting for 20.2% of variance. Tukey HSD tests showed significant contrasts: ADHD vs. ADD, ADHD vs. Concerta, and ADHD vs. Ritalin. A second one-way ANOVA revealed significant differences in FKRE scores across the seven groups (F(6, 133) = 3.69, p = .002), rejecting the null hypothesis of equal means. The effect size was moderate to large (η^2^ = 0.14, Cohen’s f = 0.41), with 14% of the variance in readability scores due to group differences. ADD content (M = 51.08, SD = 11.01) had significantly higher readability (easier to read) than ADHD (p < 0.05). No other pairwise differences (e.g., between medications) reached significance.

**Conclusions:**

ADHD-related content is significantly more difficult to read than information about ADD or medications. The lower readability of medication-related content compared to diagnostic content may prioritize pharmacological treatments over understanding ADHD or considering other interventions. This readability gap presents an opportunity to enhance accessibility and health decision-making.

**Disclosure of Interest:**

None Declared

